# Navigating the Spanish Healthcare System: Perspectives of Newcomers and Cultural Mediators on Communication, Relationships, and Fear of Deportation

**DOI:** 10.1155/jonm/4307386

**Published:** 2026-01-02

**Authors:** Francesc Ramos-Roure, Maria Feijoo-Cid, Rosa García-Sierra, Eduard Moreno-Gabriel, Clara Flamarich-Gol, Maria Almazán Gómez, Pere Toran-Monserrat, Maria Isabel Fernández-Cano, Sergio Martínez-Morato

**Affiliations:** ^1^ Department of Medicine, Faculty of Medicine, Universitat Autònoma de Barcelona, Bellaterra, Barcelona, Spain, uab.cat; ^2^ Serraparera Primary Care Center, Institut Català de la Salut, Cerdanyola del Vallès, Barcelona, Spain, gencat.cat; ^3^ Grup de REcerca Multidisciplinar en SAlut i Societat (GREMSAS), (2021SGR1484), IDIAP-UAB, Mataró, Barcelona, Spain; ^4^ Department of Nursing, Faculty of Medicine, Universitat Autònoma de Barcelona, Bellaterra, Barcelona, Spain, uab.cat; ^5^ Fundació Institut Universitari per a la Recerca a l’Atenció Primària de Salut Jordi Gol i Gurina (IDIAPJGol), Unitat de Suport a la Recerca Metropolitana Nord, Mataró, Barcelona, Spain; ^6^ Sant Roc Primary Care Center, Institut Català de la Salut, Badalona, Barcelona, Spain, gencat.cat; ^7^ Department of Medicine, Faculty of Medicine, Universitat de Girona, Girona, Spain, udg.edu; ^8^ Germans Trias i Pujol Research Institute (IGTP), Primary Care Research Group, Badalona, Barcelona, Spain; ^9^ Vila Olímpica Primary Care Center, Parc Sanitari Pere Virgili, Barcelona, Spain

**Keywords:** cultural competence, culture, emigrants and immigrants, health communication, nurse-patient relations, primary health care, qualitative research

## Abstract

**Introduction:**

The process of caring for immigrants is influenced by different cultural elements, and patients and health professionals perceive it differently. The literature often uses the concept of “immigrant patients” as if it comprises a homogeneous and nondiverse group. In this study, we focus on newcomers, since the number of years an immigrant has spent in a country conditions their experience as a patient. Despite being a more vulnerable population, there are few studies on the perspective of newcomers. Given this gap, we aimed to explore the relationship of newcomers with Spanish healthcare professionals and whether the care provided is sensitive and culturally competent from the perspective of newcomers and cultural mediators.

**Materials and Methods:**

Qualitative study with a socioconstructivist approach based on 18 interviews with newcomers and two focus groups (one with newcomers and one with cultural mediators). Purposive sampling was used to recruit newcomers, while cultural mediators were recruited by convenience sampling. The Charmaz method was applied for data analysis.

**Results:**

Newcomers and mediators point out the cultural insensitivity of health systems in their discourse, clearly expressed by newcomers’ fear of deportation, the perception of standardized care, and the prevalence of formal communication styles. For newcomers, the fear of being deported as an undocumented immigrant is a major reason for not using the health system. Newcomers and cultural mediators agreed that nursing care is standardized but disagreed about cultural issues.

**Conclusions:**

This study detected a fear of deportation and the perception of standardized care when navigating an insensitive healthcare system. Due to growing inequalities, there is an urgent need for the critical self‐reflection of the entire health system. Specific interventions, such as developing cultural competence training, revising communication protocols, and including cultural mediators on care teams, are critical to including newcomers’ needs in care practice and reducing inequalities.

## 1. Introduction

Since the 1990s, Spain has been a destination for immigrants [1]. In 2022, the immigrant population residing in Spain represented 11.62% of the population. The Spanish General Health Law establishes that public bodies are responsible for managing and providing health services and that access to these services must be universal and cover the entire population [2].

Various cultural elements influence the process of caring for immigrants and are perceived differently by the people involved, generating misunderstandings, mistrust, and distancing [3–5]. Immigrants report receiving care lacking in empathy, courtesy, and respect, while healthcare professionals describe experiencing difficulties with language, the concept of health and illness, and the beliefs of each culture [5, 6]. This mutual incomprehension causes stress and frustration in both the patient and the healthcare professional and affects the provider’s cultural sensitivity [7]. Cultural sensitivity in healthcare implies the provider being aware of the patient’s culture and how it affects their relationship, as well as being aware of their own biases to adapt care to each individual and their context [8]. The lack of respect and reciprocal trust limits the professional’s ability to care and the patient’s willingness to follow a health plan, which influences care quality and increases morbidity, mortality, and inequalities. Although cultural mediation facilitates communication, conciliation between cultures, and recognition of the other, and helps create a safe atmosphere, it is neither readily available nor accessible all the time [9].

Culturally sensitive healthcare must respond to biological, psychological, and emotional needs; offer well‐being and trust; and respect patients’ cultures, beliefs, and values [10]. Cultural competency is an ongoing process of education and willingness to care for patients from other cultures equitably, effectively, and safely within the cultural framework of the patient, their family, and their community. Most frameworks for cultural competency in healthcare are based on five pillars, offering a comprehensive approach to culturally competent and safe care: (1) cultural awareness, which involves personal introspection to recognize attitudes, prejudices, or biases toward other cultures; (2) learning about other cultures; (3) the ability to relate, communicate, and value others; (4) the motivation to learn and respect diversity; and (5) the creation of spaces for dialog and mutual understanding where experiences can be shared and cultural interaction can be strengthened [11].

Insufficient cultural knowledge, underdeveloped intercultural communication skills, and nursing and healthcare barriers are uncomfortable, thus complicating the relationship and the provision of sensitive and congruent care [12]. One review highlights the need to overcome structural barriers, improve the care relationship, and address cultural differences [13].

Most research focuses on the experiences and perceptions of cultural competency from the perspective of immigrant patients, as if “immigrant patients” were a homogenous group. The diversity within this group is reflected in differences in origin, culture, health status, number of years spent in the destination country, etc. In this study, we take the latter as the differentiating factor that conditions the patient’s experience with both healthcare professionals and the health system in general. This is why we focus on newcomers, defined as immigrants who have been in the host country for less than 5 years. Newcomers have different needs than long‐stay immigrants [14]. This is due to acculturation, a process of psychological, cultural, and social adaptation that leads to changes in values, beliefs, and attitudes [15].

Newcomers face difficulties in accessing health services and suffer from stigmatization, language barriers, and a lack of information or continuity of care. Compared to documented immigrants, undocumented immigrants use health services less frequently, and the healthcare they receive is often inadequate or insufficient [16]. This is the case with female newcomers who have a higher number of abortions and receive limited and late prenatal care [17]. For these women, maternity is far more complex due to communication barriers, difficulty accessing adequate care, and different expectations. In addition to all, this is the effort to maintain their traditions while still seeking support in a new social setting [18].

There is little research on the perspectives, perceptions, and health needs of newcomers despite being a more vulnerable population. To cover this knowledge gap, it is essential to research how newcomers experience the health system and how they interact with nurses. Therefore, this study explores the relationship of newcomers, with their unique vulnerabilities, and Spanish healthcare professionals from the perspective of newcomers and cultural mediators to determine whether the care provided is sensitive and culturally competent.

## 2. Materials and Methods

### 2.1. Design

A qualitative study with a socioconstructivist approach was conducted using 18 semistructured interviews and two focus groups with newcomers and mediators. The theoretical approach is based on the need to understand how individuals construct and interpret social reality in their daily lives [19].

### 2.2. Study Setting and Recruitment

In 2022, the immigrant population in Spain was 5.5 million (11.62% of the total population), concentrated mostly along the Mediterranean Coast and the Community of Madrid [20]. In Catalonia, the second‐most popular destination, the immigrant population is largest in major cities and the Barcelona Metropolitan area (such as L’hospitalet and Mataró) [21].

Newcomers were recruited via purposive sampling in Catalonia by (1) the primary care teams at the primary care centers of Barcelonés Nord (which covers the municipalities of Santa Coloma de Gramanet, Sant Adrià del Besós, and Badalona) and Mataró when they came for a scheduled visit with nurses, and (2) cultural mediators working for the Catalan Health System and (3) organizations offering support to immigrants in Badalona (Red Cross and Point of Newcomers of the Badalona Sur Consortium) and Santa Coloma de Gramenet (Intergramenet Foundation). Cultural mediators were recruited by the Mataró City Council using convenience sampling.

#### 2.2.1. Inclusion/Exclusion Criteria

The inclusion criteria for newcomer patients were as follows: men and women of legal age (equally distributed to achieve gender representation) from the geographic areas most prevalent at the primary care center, including North Africa, West Africa, South Asia, Central America, South America, and China; who had had at least two prior visits with nurses at the primary care center of Barcelonés Nord or Mataró; who had been in the country for less than 5 years; and who did not formally exercise the role of cultural or institutional mediator. The sole exclusion criterion was having difficulties that prevented verbal communication.

The inclusion criteria for mediators were to actively exercise the role of healthcare mediators with the communities mentioned above in Mataró. Cultural mediators are linked to the Mataró City Council and work directly with the communities included in the study. They have completed professional training, enabling them to provide mediation services.

### 2.3. Data Collection

In 2020–21, we collected and audio‐recorded 18 semistructured interviews and two focus groups: one for newcomers and one for mediators. All participants received a gift voucher to cover travel expenses.

Semistructured interviews were conducted until data saturation was reached. During interviews, cultural mediation services were available in cases where there was a language barrier or communication had to be facilitated. An interview script was outlined to assess whether the care provided was culturally sensitive and competent (based on the criteria defined in the Introduction) and included three main themes: use of the health system; experiences and expectations of visits with health professionals; and health‐related cultural aspects. The scripts for newcomers addressed topics such as reasons for migrating: positive and negative experiences with the healthcare system, in both urgent care and follow‐up of chronic illnesses; the importance of having a health card; the role of culture in healthcare; and areas for improvement. Questions such as the following were asked: “Tell us about your first experience with the Catalan healthcare system and the reason for your visit.”, “What did having or not having a healthcare card mean to you?”, “How would you describe the communication and relationship with the professional during the visit?”, “How would you have liked it to have been?”, “Describe what makes you feel good, safe, and calm when you attend check‐ups with your doctor or nurse, and why.”

The interviews lasted an average of 40 min. None of the researchers knew the study participants in advance. Six experts in research, immigration, and primary care nursing validated the interview script following the Delphi method—a structured and recursive research technique that uses multiround questionnaires to reach a consensus between experts, thus ensuring high reliability and alignment with the study aims [22]. Two rounds were conducted, applying Lashe’s test to assess consensus. A value of +1 or −1 is given based on the degree of agreement of each participant. If the total sum of the participants is positive for a specific item, it means that there is agreement among participants. If the result is negative, it means that there is disagreement on that item and the question should be asked again based on the comments made by participants [23].

After analyzing the data from the individual interviews, two focus groups were established: one formed by six newcomers (1:22 h), and the other by five cultural mediators (1:42 h). The newcomers’ focus group comprised six immigrant patients and was aimed at delving deeper into the themes that emerged from the semistructured interviews and confirming data saturation. This focus group met at a social organization in Badalona. After analyzing the results, we conducted the cultural mediators focus group. The script of this focus group was written to compare the main results. The script for cultural mediators followed a structure like that of the newcomers’ script. Questions such as the following were asked: “Based on your professional experience, what competencies should be strengthened in healthcare professionals to provide better care to newcomers?”, “Based on your experience, do you believe current care is appropriately adapted to cultural diversity?”. The focus group with five cultural mediators was held at the Primary Care Center of Mataró.

### 2.4. Data Analysis

The data were analyzed using Charmaz’s approach to grounded theory [24]. Firstly, the recorded audio data were transcribed, and the transcribed material, recordings, and field notes were classified. The transcripts were then reviewed and coded. Two researchers worked together on data coding and interpretation. Researchers started the open coding phase using the software Atlas.ti for the qualitative analysis. The process was iterative and allowed the most important themes to emerge. These were explored in greater depth as the analysis progressed, following the steps outlined by Charmaz: initial coding, focused coding, memo writing, and developing theories. The constant comparison method was applied throughout the process, making it possible to detect the most significant differences and continuously and rigorously compare the data throughout the entire process. Discrepancies between the researchers were discussed until consensus was reached in each coding phase. Lastly, a third researcher reviewed the results.

### 2.5. Ethical Considerations

The project was presented to the Clinical Research Ethics Committee for approval (P18/130). All participants signed an informed consent form. Personal data were coded using codes and pseudonyms to ensure participants’ anonymity. All the data were stored using secure methods to ensure confidentiality.

### 2.6. Rigor and Reflexivity

The researchers who analyzed the results and the one who conducted the interviews and focus groups are all nurses: One is an associate professor at the university, and the other two are primary care nurses—one of whom works in the region studied. As insider researchers, they were intimately familiar with the field of study but were also aware of the need to maintain a “critical perspective,” balancing objectivity and subjectivity [25]. Given our insider nature, we had to navigate between professional and research aspects [26]. To do so, we conducted (1) an exercise of critical awareness of our own values, beliefs, and prejudices; (2) a reflexive process of the power dynamics that could be established in our relationship as researchers and professionals with the study participants; (3) at the epistemological level, a continuous and self‐critical reflexive process on various aspects of the study, from the methodological approach and data collection instruments to the subsequent analysis and interpretation of the results. Lastly, the COREQ checklist for qualitative studies was used as an instrument to ensure quality, transparency, and methodological rigor and the interpretation of the results [27, 28].

We followed Lincoln and Guba’s criteria (1985) to improve study rigor. Multiple strategies addressing credibility, transferability, reliability, and confirmability were employed. Credibility was strengthened through the use of an interview guide reviewed by experts, which guaranteed the purpose of the study would be covered. Moreover, by including the perspectives of both newcomers and cultural mediators, we used triangulation to compare and contrast perspectives. To strengthen confirmability, one researcher who was not involved in data collection or analysis reviewed the analysis and coding process and discussed the results from an outside perspective. Reliability was enhanced by the accuracy and description of data collection. To avoid interpretations from the cultural background of the researchers, the results were reviewed by a checking member of each group (a patient and a mediator). The analysis and quotes of the participants that illustrate the main themes helped with the consistency of the findings.

## 3. Results

The mean age of newcomer participants was 38. They came from 15 countries and had been living in Spain for 2 years on average. The mean age of cultural mediators was 50. They had been living in Spain for 23 years on average (see Tables 1 and 2).

**Table 1:** Sociodemographic data of the participants.

Number	Participant	Code	Technique	Sex	Age	Country of origin	Years in Spain	Educational level	Geographical area
1	Patient	E1	Interview	Woman	30	Nigeria	2	Secondary school	West Africa
2	Patient	E2	Interview	Woman	35	Honduras	3	Primary school	Central America
3	Patient	E3	Interview	Woman	51	Peru	4	Secondary school	South America
4	Patient	E4	Interview	Woman	34	Peru	1	Secondary school	South America
5	Patient	E5	Interview	Woman	28	Colombia	2	Secondary school	South America
6	Patient	E6	Interview	Woman	42	Honduras	2	Secondary school	Central America
7	Patient	E7	Interview	Man	18	Mali	1	Primary school	West Africa
8	Patient	E8	Interview	Man	38	Morocco	2	Primary school	North Africa
9	Patient	E9	Interview	Man	47	Morocco	1	Primary school	North Africa
10	Patient	E10	Interview	Man	37	Morocco	1	Primary school	North Africa
11	Patient	E11	Interview	Woman	39	Equatorial Guinea	2	Secondary school	West Africa
12	Patient	E12	Interview	Woman	33	El Salvador	3	Secondary school	Central America
13	Patient	E13	Interview	Woman	30	Pakistan	1	Secondary school	South Asia
14	Patient	E14	Interview	Man	43	Gambia	2	No schooling	West Africa
15	Patient	E15	Interview	Man	32	Gambia	2	No schooling	West Africa
16	Patient	E16	Interview	Woman	42	Morocco	1	No schooling	North Africa
17	Patient	E17	Interview	Woman	32	China	3	Secondary school	China
18	Patient	E18	Interview	Man	25	China	3	University	China

19	Patient	GF1	Focus group	Man	41	Morocco	3	Secondary school	North Africa
20	Patient	GF2	Focus group	Man	43	Senegal	3	University	West Africa
21	Patient	GF3	Focus group	Woman	47	Honduras	4	Secondary school	Central America
22	Patient	GF4	Focus group	Woman	83	Venezuela	1	Secondary school	South America
23	Patient	GF5	Focus group	Man	28	Morocco	2	Secondary school	North Africa
24	Patient	GF6	Focus group	Man	48	Egypt	2	Primary school	North Africa

25	Cultural mediator	MC1	Focus group	Woman	47	Gambia	37	Secondary school	West Africa
26	Cultural mediator	MC2	Focus group	Woman	50	Romania	20	University	Europe
27	Cultural mediator	MC3	Focus group	Woman	55	Peru	13	University	South America
28	Cultural mediator	MC4	Focus group	Woman	56	Morocco	29	University	North Africa
29	Cultural mediator	MC5	Focus group	Woman	41	Senegal	14	Secondary school	West Africa

**Table 2:** Description of participants.

Participants	Interviews	Focus group
Recently arrived immigrants	18	6
Age (mean)	35	48
Women	11	2
Men	7	4
Years in Spain (mean)	2	2.5
Cultural Mediators		5
Age (mean)		50
Women		4
Men		1
Years in Spain (mean)		22.6

### 3.1. Navigating an Insensitive Healthcare System

**Figure 1:**
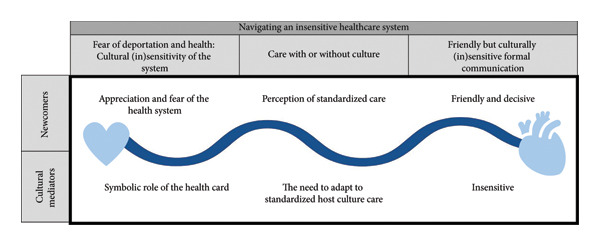
Navigating an insensitive healthcare system.

Both newcomer patients and cultural mediators reported fear when navigating the healthcare system, as well as the lack of sensitive and culturally competent care. We have thus highlighted the fear of deportation and perception of standardized care when navigating an insensitive healthcare system as the main category. Three coconstructed subcategories grounded in the data yielded this main category: (1) fear of deportation and health: cultural (in)sensitivity of the system; (2) care with or without culture; and (3) friendly but culturally (in)sensitive formal communication (see Figure 1).

To better distinguish information obtained from newcomers’ discourse from that of cultural mediators, we have created two sections for each of the aforementioned categories.

### 3.2. Fear of Deportation and Health: Cultural (In)sensitivity of the System

The search for safety during the migratory process does not end when newcomers arrive in the destination country; instead, it gets transformed into fear when they fall ill and believe that accessing the health system may be cause for deportation.

#### 3.2.1. Newcomers: Appreciation and Fear of the Health System

Most immigrant patients report that the Catalan health system meets or exceeds their expectations due to its universality and the care being more satisfactory than in their country of origin. However, this appreciation comes with ambivalence. While healthcare is related to safety, accessing it triggers fear of being deported. In this phase, healthcare is sometimes feared, delayed, and avoided.

The treatment I receive is beyond what I request and expect. (E10)

Sometimes, you think you’re really ill, and you say: “I’m not going because I′m afraid they’ll deport me.” So, you endure illness out of fear. (E2)

In that sense, while Spanish legislation grants all people access to the health system, including undocumented immigrants, participants described barriers that reveal systemic inconsistencies. For example, it is not always easy to get registered in the municipal census, which is a prerequisite to obtaining the health card—a personal document that identifies citizens as users of the public healthcare system. These bureaucratic obstacles contribute to a perception of exclusion and reinforce fear. People without a health card are not eligible to access chronic or specialized healthcare services (DOGC, 9/2017, 27 de juny). For many, obtaining a health card is a turning point.

Nobody wants to register you so you can get the health card. You have to ask for it, sometimes even pay for it. When I got it, I felt happy. (E2)

#### 3.2.2. Cultural Mediators: Symbolic Role of the Health Card

Cultural mediators observe that newcomers feel insecure when they do not yet have their health card. They try to reassure them by explaining that they have the right to assistance in the event of a health problem. Obtaining this card is comparable to achieving a major goal, dispelling fears, and transiting from invisibility to participation in society. According to the mediators, the health card helps immigrants root themselves in the host country and is part of developing an identity in their new home, highlighting its symbolic role in the integration process.

The card means security if something happens, to go to the hospital or the primary care center without trouble. It is also part of the identity process for those here, […] It is a link. (MC4)

### 3.3. Newcomers’ Perception of Standardized Care: Care With or Without Culture

Newcomer patients believe that professionals are responsible for addressing health‐related cultural issues. Those who did not place value on culturally sensitive care pointed out several reasons for their stance. For the youngest, it is because they maintain only an occasional relationship with the health system based on emergencies and thus cannot value the inclusion of culture. For others, it is because healthcare professionals solve their health issues with friendly care despite not understanding their culture, and that is enough; cultural adaptation is not necessary. Some participants did not value culture because they understood the system to be an alternative outside their cultural framework that they resort to when self‐care does not suffice.

If someone [the professional] doesn’t ask you about culture, it’s for a reason, because that’s not what they’re there for (E11).

I cannot judge people in a 20–30 min visit; I have not had continuous contact with them to know if they really understand and respect my culture and religion. (E14)

I try to take natural medicines, herbs, to feel a bit better and not go to the doctor. (E12)

They report that some professionals ask about more obvious or commonplace issues, such as why they wear a veil or the reasons for certain dietary restrictions. However, they make no extra effort to learn more or understand their culture and how it influences their care. The newcomers point out three aspects that show their culture has been considered: food, religion, and language. Muslim newcomers report that their religion and associated dietary restrictions were taken into account. Some Latin American newcomers emphasize that when professionals speak to them in Spanish, it is a sign of cultural respect and adaptation to their Latin culture (since in Catalonia, both Catalan and Spanish are spoken).

They are surprised you don’t drink alcohol, don’t smoke… (GF2)

Sometimes they ask why we wear a veil, and we say yes, we are going to wear a veil because it is our religion, we have to cover our hair, and so on. Then she says there is no problem. (E13)

When they speak to me in Spanish, I feel like they are trying not to make me feel inferior, they are letting me feel my culture. (E2)

Besides these general, more apparent aspects, care is not adapted to culture. For most newcomers, as well as for cultural mediators, care is standardized.

They usually treat you like a sick person and that’s it, they do their job, which has nothing to do [with culture]. (E8)

#### 3.3.1. Cultural Mediators: The Need to Adapt to Standardized Host Culture Care

For most newcomers, as well as for cultural mediators, care is standardized.

[About healthcare] It’s generic. It just is. For you, for you, and for you. (MC2)

Intercultural mediators, however, see culture as present whenever there is disagreement between professionals and newcomers. They report that the tendency during visits with healthcare professionals is to hold the immigrant responsible for discrepancies. That is, the immigrant must constantly adapt to the host culture. They highlight the need for health centers to act as a vehicle for the integration and acculturation of immigrants in the community.

It is always the immigrant who has to adapt, the immigrant who has to hold the knowledge… The immigrant who has to go through reception and… Everything… They have to do everything. (MC4)

Fifteen years ago… we trained all the patients who came to the primary care center. […] Mediators. Groups of women, men, work with children. We explained nutrition […] Pregnancy… Contraceptive methods… Breastfeeding… […] All that used to be done. Now […] nothing is done. […] There is no time, no funding… (MC3)

### 3.4. Friendly But Culturally (In)sensitive Formal Communication

Communication is key, as it influences newcomers’ trust in the health professional as well as their perception of the care offered.

#### 3.4.1. Friendly and Decisive for Newcomers

Newcomers consider communication an essential component in healthcare relationships. They are generally satisfied with the care they receive from the primary care team, believing it to be of good quality (it provides a solution to the health problem) and that they are treated excellently. Newcomers most frequently highlight the healthcare professionals’ friendliness, although they also identify politeness, respect, patience, and empathy. Part of feeling treated well is being included in developing their healthcare plan, or rather, when the nurses allow them to express their opinions and concerns during the visit.

From the moment I arrived at the Primary Care Center, they have been very kind and patient (E3)

You feel that both the doctor and the nurse want you to participate. But that depends on the communication between the patient and the doctor. (E2)

They define competent communication as establishing an informal, fluid, and natural interpersonal dialog in which information and knowledge are exchanged, and emotions, concerns, needs, and feelings can be expressed. Informal conversation and active listening during visits are highly valued. However, nurses’ communication is formal, polite, and satisfactory enough to resolve the reason for the visit. Patients find that some nurses do not possess adequate communication and listening skills or motivation and that they should improve their communication with patients.

Dialogue is very important because it frees the person, and the patient feels trust… like… a state of trust where they tell you everything. (GF2)

Communication is dialogue: I like it when I talk to them, and they listen to me, advise me, pay attention to what I’m explaining, and understand me well. (E11)

Newcomer patients are initially quite satisfied with the information provided by professionals, although as their relationship with the health system progresses, they find it does not meet all their needs. They require more extensive and detailed information because they believe it forms part of quality patient care, deepens trust in health professionals, and is aligned with their expectations.

For them to explain things to you in detail, and not just tell you “you’re fine” and that’s it. (E4)

In general, newcomers do not mention racist, discriminatory, or authoritarian attitudes among healthcare personnel. They find that healthcare professionals offer humane, fair, and dignified treatment to all, regardless of their origin, and that equal treatment is a sign of cultural respect.

They have never treated me differently because of my race or skin color. On the contrary, that smile they give you is the most important thing to me. (E9)

They respect me: they show it by being pleasant and charming, they show interest and care. (E1)

#### 3.4.2. Insensitive for Mediators

Cultural mediators often find that patients have not understood all the information provided or need the mediator to reiterate it. They acknowledge that this can only be resolved with more involvement from health professionals.

When I’ve accompanied patients… I’ve left and had to sit somewhere and explain what the doctor said again. But calmly. “And what is this for? And what’s that? And what did he say? I don’t remember.” (MC3)

Cultural mediators disagree with newcomers, pointing out that discriminatory attitudes and experiences of poor treatment are recurrent in the care newcomers receive. It is worth noting that the opinions expressed by newcomers are based on their personal experiences, whereas cultural mediators have more experiences.

“She just gave birth. She went to the doctor, and I went with her to the checkup. And they said: “No, no, only the mother and child can come in.” And they thought I was the mother because the child was white. […] “No, no, I’m not the mother, she is.” “How is that possible, the child is white. And his father?” […] Can we see your documentation?” (MC3)

## 4. Discussion

This study helps expand the literature from the perspective of immigrants who use the Spanish health system shortly after arriving in the host country. Newcomers and cultural mediators report a fear of deportation and a perception of standardized care when navigating an insensitive healthcare system. Newcomers’ fear of deportation until they are documented is a major reason for not using the health system. They do not believe cultural issues need to be considered and agree with mediators that care is standardized. However, mediators always identify cultural differences in conflicts. Both newcomers and mediators consider communication an essential component that should be based on informal conversation and active listening, despite the relationship with the nurse being formal and polite enough to solve the health problem, albeit standardized.

The evidence shows that undocumented immigrants (many of them newcomers) describe fear and insecurity as the primary emotions they experience when they need to use the health system. The fear of undocumented immigrants (newcomers or otherwise) of being detained and/or deported is universal [29, 30]. When immigrant women use the health system for issues related to pregnancy, maternity, and child care, they also feel constant anxiety and fear of not being able to protect their children, whether newborns or older children [31].

This fear of deportation, which leads immigrants not to use the system when they experience health problems, is an example of the (in)sensitivity of the system. Despite international agreements on human rights regarding access to healthcare, they are not applied in all countries, especially in the case of undocumented immigrants [32]. From one country to the next, there are significant differences in terms of the healthcare provided to undocumented immigrants, even within the EU [32]. Most countries do provide emergency healthcare, although not necessarily always for free. Few countries offer essential health services beyond emergency care, despite the growing evidence from political economy analysis and the manifold recommendations to change legislation and provide full health coverage to the immigrant population regardless of their administrative status [30]. Institutions must review protocols and ensure confidentiality to create “safe spaces” where patients feel protected regardless of their administrative status, especially in countries with restrictive immigration policies [33, 34]. Different countries take different approaches to the challenges newcomers face in the healthcare system. In Australia and New Zealand, culturally safe care policies, professional diversity, and improved communications are prioritized [35]. The Multicultural Health Action Plan 2023–2027 includes cultural competence as a core value and promotes self‐assessment, the institutionalization of cultural knowledge, and the adaptation of institutions [36].

Cultural safety in health is an approach aimed at helping professionals and institutions recognize how their own culture, values, and prejudices influence the care they provide. It requires ongoing critical self‐assessment to identify biases, reflect on power, and assume responsibility for transforming it. The goal is to create environments where people feel respected and valued, ensuring equitable care that removes barriers and promotes health justice [37]. A culturally safe space is one where people feel heard, respected, and valued, and where their identity, culture, experiences, and needs are integrated into care. It is an environment in which healthcare professionals and institutions examine their own biases, power dynamics, and structures to provide safe care from the patient’s perspective [37, 38]. This approach shifts control from the provider to the patient, promoting their active participation and equity and respect in healthcare [38, 39].

There are several examples of the implementation of cultural safety policies in health. In Canada, since 2015, the Declaration of Commitment and the Cultural Safety and Humility Committee have ensured the integration of cultural safety at all levels of the health system [40]. In Australia, the Aboriginal Cultural Security Policy recognizes Aboriginal cultural heritage, promotes the inclusion of Aboriginal health personnel, improves communication, and applies cultural security in all areas of the health system [41]. At Katherine Hospital in Australia, Indigenous culture and cultural determinants of health were incorporated into clinical practice, combining Aboriginal and Torres Strait Islander cultural practices with biomedical knowledge, and allocating resources to ensure culturally safe care [42]. With due regard for the differences, these types of policies and commitments to providing cultural safety for the Indigenous population and ethnic minorities in countries such as Australia, New Zealand, and Canada could serve as a model for adapting to newcomers. Applying them in our context would help create safe spaces that foster trust and reduce the fear of deportation.

At the level of professionals, healthcare providers must understand and manage patients’ concerns about immigration status, language barriers, and precariousness [43].

Although the impact of cultural competency training varies, it can raise staff awareness, reduce bias, and improve the quality and safety of care [44, 45]. The most effective programs combine different educational strategies and simulation, showing improvements in competence and empathy [46–49]. There is evidence for how digital health technologies can facilitate access; although if poorly designed, they aggravate the digital divide and decrease access [50–52]. If they are adapted to cultural and linguistic diversity, with the active participation of immigrant communities, they improve digital literacy and care, as shown by digital programs in Afghan immigrants with anxiety and depression [50, 53, 54].

On the other hand, as seen in our results, newcomer patients believe that professionals are responsible for addressing cultural aspects related to health, despite not delving into how culture conditions care in the relationship between professionals and users. The literature reveals how important it is for healthcare professionals to develop cultural competency, given the ever‐growing cultural diversity they face. It also promotes a model that benefits from including patient‐centered care, with active involvement to bring care closer to the community [55]. Although this framework is well‐established, it presents challenges in practice. It has been noted that short training workshops rarely produce lasting changes in behavior or institutional culture [56]. Additionally, inconsistencies in theoretical approaches and training methods make it difficult to assess how such training impacts patient outcomes [57]. It should be noted that there have been research involving training initiatives in combination with the implementation of supportive evidence‐based guidelines in Europe, showing that the combination of methods can enhance cross‐cultural communication, understanding and trust between practitioners and migrant patients [58]. Some innovative proposals also show promising results in improving cultural competence, such as the study by Moreno‐Comellas et al. [59], which designed an intervention using an escape room targeted at healthcare professionals in Barcelona. The activity enabled participants to experience and reflect on challenges faced by migrants, leading to a positive impact on the development of their cultural competence. Moreover, simulation‐based learning in healthcare students has shown promising results and opens an innovative avenue for developing cultural competencies and awareness among healthcare professionals [49, 60]. A systematic review evaluating educational interventions for nurses shows that cultural competencies, health disparities, and awareness can be improved through learning from discussion groups, reflection sessions, presentations, and case studies [48].

Culturally sensitive healthcare requires going beyond the traditional notion of acquiring knowledge about “other” cultures. The concept of cultural humility represents a dynamic and ethical alternative that focuses on self‐reflection, acknowledgment of power imbalances, and horizontal relationships with the communities served [61]. In practice, it is suggested that this approach replace the “competency” approach, both at the curricular level of health professionals and structurally in social initiatives and policies. Some potential paths for moving toward this possibility involve making health a cross‐cutting parameter in the legislation of other areas equally relevant to newcomers, such as education, housing, or other social services [34]. This could help promote a willingness to listen and cocreate meanings, which is more consistent with the principles of person‐centered care [62].

A study by Lauwers et al. [63] suggests that people cared for in diverse contexts value it when their diversity is taken into account as a constituent part of fair and respectful care. In this context, some participants do not value culture in care, believing that (a) it is not necessary, (b) it is not possible to value it if the relationship is not maintained over time, and (c) professional care is an alternative outside the patient’s cultural sphere that should be resorted to when self‐care measures do not suffice. One could argue that understanding primary care services in this way forms part of integrating the values inherent to the use of health services in the host country [64].

For most newcomers and cultural mediators, care is standardized and only slightly or not at all adapted to everyone’s cultural context. To them, the patient must adapt to the healthcare setting. According to some, this can be interpreted as a sign of cultural assimilation, based or not on a bias from professionals [64]. All these phenomena are part of the acculturation process, which the literature portrays as a process for which it is challenging to draw conclusions [65]. In any case, it is relevant that the need for continuous care may condition how users perceive care to be influenced by culture. Continuous care results in more interactions where cultural discrepancies may appear, according to the mediators in our study and long‐stay immigrants in the host country [66]. Regarding these long‐term experiences, it may be interesting to encourage racial and ethnic diversity among healthcare workforces to contribute to the design and implementation of interventions mitigating immigration‐related stressors [34].

The negative experiences perceived by the participants are similar to the unequal treatment described by other authors in power relations related to health culture, regardless of the patient’s origin [67]. It is worth emphasizing the unequal power relationship already inherent to interactions with immigrants, often underscored by communication barriers [4]. Some publications show a negative association between discriminatory and stereotypical attitudes of professionals and the professionals’ communication styles, misunderstandings, nonverbal communication, time devoted to the patient, treatment recommendations, and trust [66, 67].

Communication is an essential element in quality care for newcomers, who define competent communication as the informal, respectful, and two‐way dialog between patient and health professional, which facilitates emotional expression and satisfies the patient’s information needs [68]. This definition is in line with that of culturally sensitive communication, or the verbal and nonverbal interaction between individuals from different cultures, adapted to the characteristics and preferences of each person, with the aim of developing collaboration strategies with the patient and family, and providing equitable and culturally sensitive care through mutual understanding and respect [69].

The study shows that nurses use a formal communication style during patient visits, offering kindness and respect but not meeting other needs mentioned by patients and cultural mediators. Formal communication is cold, systematized, inflexible, and neglects some social aspects [70]. More formal language could be interpreted from an objective perspective as treating “everyone equally” based on the hegemonic culture and asymmetric relationships that do not consider individual differences or perspectives [71]. Informal conversation should be the standard communication style since it is natural and flexible in different cultural contexts and situations. It promotes satisfactory encounters, acceptance of the other, and a comprehensive view of the patient, their family, and their setting, including their health needs, concerns, and expectations [70]. Renshaw describes dialog as an opportune practice to exchange information, share meanings, values, and beliefs and thus create a shared world vision to achieve consensus, harmony, and mutual understanding [72]. Dialog is an intrinsic characteristic of human beings and part of the social process. To perceive and understand, you must first talk [72]. Furthermore, conversation is an iterative process prescribed to explore, learn, and transfer knowledge dialogically [72]. In dialogic learning, interlocutors gain new knowledge by reaching a consensus through egalitarian dialog, overcoming homogenization, and respecting differences, leading to the ability to transform reality [71].

### 4.1. Strengths and Limitations of the Work

The study sample was limited given the origin of participants (newcomers) and difficulty in accessing them (due to their high vulnerability, migratory stress, etc.), which restricts the transferability of the data. However, we provide evidence by comparing the perspectives of newcomers and cultural mediators. Future research should delve further into this field. Maximum heterogeneity and representativeness were sought out in the participant groups to collect the maximum number of opinions. Many participants were young or middle‐aged and had little experience in their native health system since they rarely had chronic conditions or used primary care services. To curtail the impact of this limitation and provide depth and greater plurality to the data collected, most newcomers were recruited through associations that host newly arrived immigrants. There, older users were sought out since it was more likely they would have experienced more continuous care with nurses and had greater experience with the health system. Language barriers were overcome using cultural mediators, assuming the potential risk of bias in interpreting the data. However, given the limited literature on this population, more research should be conducted in this field. We did not take “years of experience as a cultural mediator” into account as selection criteria due to the difficulty in recruiting them and the shortage of mediators. This limitation might influence both the way cultural mediators view and interpret communication and their perception of how culture influences communication.

## 5. Conclusion

Newcomers and mediators reflect the cultural insensitivity of health systems in their discourse, clearly expressed by newcomers’ fear of deportation, perception of standardized care, and the prevalence of formal communication styles. Addressing the inequalities experienced by newcomer patients requires collaborative interventions at multiple levels between patients, families, healthcare professionals, healthcare systems, and policymakers/politicians. We suggest that, on an individual basis, healthcare professionals implement interventions to increase their cultural knowledge and critical awareness of how the social and political context shapes identity (including that of healthcare professionals and patients) [73] and how these factors influence their interactions with patients [74]. We recommend reviewing communication protocols and including a cultural formulation interview as recently included in the DSM‐V, since it is a tool that facilitates the integration of patients’ backgrounds and beliefs into care provision and (b) training programs aimed at increasing cultural sensitivity.

At the community level, to increase immigrant participation in formal health settings, (1) more cultural mediators should be incorporated into primary care centers; (2) cultural mediators should be included on care teams when carrying out community interventions; (3) newcomers should be offered courses/talks on how to safely navigate the system (to lessen the fear of deportation) and thus become an agent and actor in the development of initiatives to promote inclusion.

At the organizational and structural levels, health systems must move away from insensitivity and become “immigration‐informed” and “immigrant‐friendly,” that is, health systems with a focus on the physical and psychological safety of immigrants. This requires organizational strategies such as (1) implementing interventions to address health literacy barriers for newcomers; (2) coparticipating with communities to create/define/enable the role of peer healthcare navigators—immigrant men and women who explain how to navigate the healthcare system (to lessen the fear of deportation); (3) professionalizing the role of the cultural mediator; establish a cultural mediator job pool for healthcare settings; and (4) establishing ties with existing community associations and immigrant rights and services organizations.

It would be valuable to explore this line of research further. Studies exploring the outcomes of cultural competence training on the attitudes and practices of healthcare professionals are needed. It would also be beneficial to examine how immigrants’ experiences in healthcare vary across various settings (e.g., public vs. private healthcare) and cultural contexts. Examining the role of technology in enhancing cultural competence (e.g., telemedicine or language translation tools) could be a fruitful area for exploration as well.

## Conflicts of Interest

The authors declare no conflicts of interest.

## Funding

This work was supported by the Col·legi Oficial d’Infermeres i Infermers de Barcelona (PR‐366/2019).

## Data Availability

The data that support the findings of this study are available from the corresponding author upon reasonable request.
